# The Extremely High Adsorption Capacity of Fluoride by Chicken Bone Char (CBC) in Defluoridation of Drinking Water in Relation to Its Finer Particle Size for Better Human Health

**DOI:** 10.3390/healthcare6040123

**Published:** 2018-10-10

**Authors:** H. M. Ayala S. Herath, Tomonori Kawakami, Masamoto Tafu

**Affiliations:** 1 Department of Environmental Engineering, Faculty of Engineering, Toyama Prefectural University, 5180 Kurokawa, Imizu, Toyama 939-0398, Japan; kawakami@pu-toyama.ac.jp; 2 National Institute of Technology, Toyama College, 13 Hongo, Toyama 939-8630, Japan; tafu@nc-toyama.ac.jp

**Keywords:** adsorption, carbonizing, particle size, isotherm, ion exchange, fluoride

## Abstract

The ingestion of fluoride-contaminated water causes serious health issues in people all over the world. In the current study, the adsorption of fluoride onto chicken bone char (CBC) was investigated as a defluoridation technique. Finer-sized CBC with a diameter of 106–212 µm was used to investigate the fluoride adsorption capacity onto CBC. Results revealed that finer-sized CBC yielded an unusually high fluoride adsorption capacity of 11.2 mg/g at the equilibrium fluoride concentration of 10 mg/L. The study shows that CBC can be utilized in the defluoridation of drinking water and that finer-sized CBC enhances ion exchange to perform a higher adsorption capacity.

## 1. Introduction

Fluoride is widespread in the geological environment [1], and fluoride commonly occurs as fluorspars (CaF_2_), cryolite (Na_3_AlF_6_), and fluorapatite (Ca_10_(PO4)_6_F_2_). The fluorspar mineral is found in sedimentary rocks, and the cryolite mineral is found in igneous rocks [2]. The dissolution of fluorine-containing rocks releases fluoride into groundwater [1]. Industrial effluents that contain high amount of fluoride discharged into groundwater cause contamination of groundwater [2]. Groundwater is the main source of fluoride ingestion into the human body [1] in places where people use groundwater for drinking and cooking [3].

The World Health Organization (WHO) guideline for fluoride in drinking water is less than 1.5 mg/L [4]. Excess fluoride concentrations in groundwater have been reported in more than 20 developing and developed countries [5]. It has been estimated that 260 million people in the world are consuming drinking water with a fluoride concentration of more than 1.0 mg/L [6]. The fluoride concentration in drinking water is an important factor when determining water quality for human consumption. The ingestion of fluoride into the human body will either be beneficial or detrimental based on its concentration [7]. Fluoride is an important microelement for keeping human bones and teeth in a healthy condition [8]. However, an excess amount of fluoride intake causes human health hazards, such as dental fluorosis and skeleton fluorosis [9]. Furthermore, fluoride has been named as one of the suspicious causes of chronic kidney disease of unknown etiology (CKDu), causing a number of deaths in Sri Lanka [10]. Therefore, treatment of fluoridated water is essential and one of the paramount environmental concerns in the world [11].

Several defluoridation techniques are used worldwide in order to remove fluoride from contaminated water, such as precipitation [12], coagulation [13], adsorption [14], ion exchange [8], reverse osmosis [15], nanofiltration [16], electrolysis [17], and electrodialysis [18]. However, access to most of these techniques is limited due to the high operational cost and the formation of sludge. Among the techniques mentioned above, adsorption is a widely used method of defluoridation due to its cost effectiveness and ease of operation [17]. When considering adsorption techniques, the adsorption of fluoride onto bone char is a highly effective and cost-effective technology that can be used in defluoridation [19]. Defluoridation of drinking water by bone char is an old, enduring technology [20].

In this study, our attention was focused on fluoride removal by finer-sized chicken bone char (CBC) particles in relation to its adsorption capacity.

## 2. Materials and Methods

### 2.1. Preparation of Chicken Bone Char (CBC)

Three temperatures were selected to determine the best carbonizing temperature for preparing CBC. The chicken bones were carbonized under an anaerobic condition in closed metal containers by an electrical muffle furnace with temperatures of 673 K, 873 K, and 1073 K for 1.5 h in order to prepare CBC. The prepared CBC was washed with clean water several times and dried at 378 K in an electrical oven for one night.

CBC with a diameter of 106–212 µm was selected to investigate the effectiveness of defluoridation by finer-sized CBC. The CBC was hand-crushed in a dry condition using a mortar and pestle and then hand-sieved using 106 µm and 212 µm sieves to prepare the particles with a diameter of 106–212 µm. This was the minimum possible particle size with a high adsorption capacity in the defluoridation of drinking water as particles with a diameter less than 106 µm is impossible to prepare on a large scale.

### 2.2. Equilibrium Sorption Isotherms

Isotherm studies were conducted for each set of carbonized CBC at 673 K, 873 K, and 1073 K to investigate the adsorption capacities of CBC at different temperatures. Synthesized drinking water with a fluoride (F^−^) concentration of 10 mg/L was prepared using sodium fluoride (NaF) and deionized water for the isotherm studies. Each set of CBC with a diameter of less than 45 µm was shaken with weights of 0.01 g, 0.02 g, 0.03 g, 0.04 g, 0.05 g, 0.1 g, 0.15 g, and 0.2 g and 50 mL of prepared F^−^ solution in 50 mL vials in an electrical shaker with a 180 reciprocation. After 24 h of shaking, samples were taken from each vial and filtered through a membrane filter with a pore size of 0.45 µm to remove any suspended CBC. The fluoride concentration of each sample was measured with an ion selective electrode (ORION STAR A324 pH/ISE Meter and ORION 9609BNWP Ionplus Sure-Flow Fluoride Electrode). The Total Ionic Strength Adjustment Buffer (TISAB III) was added to each sample before analysis to avoid interference during measurements.

One of the three sets of carbonized CBC at three different temperatures with the highest fluoride adsorption capacity was selected for further experiments.

Isotherm-analyzing studies were conducted to investigate the effect of Cl^−^ on the adsorption capacity of the CBC because high concentration of Cl^−^ was detected in the final solution after F^−^ adsorption. Synthesized drinking water with a fluoride concentration of 10 mg/L and Cl^−^ concentrations of 0 mol/L, 0.01 mol/L, 0.1mol/L, and 1.0 mol/L were prepared by dissolving NaF and NaCl into deionized water. The CBC with a diameter of 106–212 µm was shaken with weights of 0.03 g, 0.04 g, 0.1 g, 0.2 g, 0.3 g, and 0.5 g and 50 mL of the solution in 50 mL vials in an electrical shaker with a 150 reciprocation. After 48 h of shaking, the fluoride concentration was measured in the same manner as previously mentioned.

### 2.3. Fluoride Removal by CBC Column Experiment

**Figure 1:**
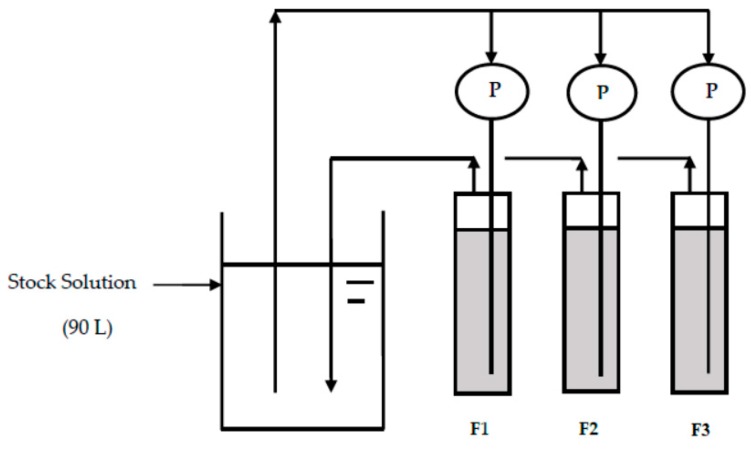
Schematic diagram of the fluoride removal setup.

Figure 1 shows the schematic diagram of the fluoride removal setup by CBC. Three plastic vessels (F1, F2, and F3) with a bed height of 8 cm and a diameter of 6.5 cm were used for the experiment. Fifty-five grams of CBC with a diameter of 106–212 µm was placed into each column. Synthesized drinking water with a fluoride concentration of 20 mg/L (stock solution) prepared by sodium fluoride (NaF) and deionized water was allowed to pass through each column with a flow rate of 2.4 L/day via a pump (P). The outlets of the F1, F2, and F3 columns were connected to the bucket for the stock solution. The three plastic vessels (F1, F2, and F3) with a bed height of 8 cm and a diameter of 6.5 cm were changed into three glass columns (F1, F2, and F3) with a bed height of 21 cm and diameter of 3 cm after 20 days of operation for a uniform flow. The same amount of CBC was placed into the glass columns, and the same flow rate was maintained. The fluoride concentration of the stock solution and the outlets were regularly measured using the ion selective electrode. After 48 days of operation, the fluoride concentration was found to have decreased to a level below 10 mg/L, and NaF equivalent to the concentration of 10 mg/L was therefore added to the stock solution. F^−^ was added to the solution at days 58, 106, 130, 132, 139, and 143 using NaF to keep the F^−^ concentration around 10 mg/L. The pH of the solution was measured using a pH meter (ORION STAR A324 pH/ISE Meter and Beckman Electrode 511070).

The synthesized fluoride solution was allowed to pass upward through each column to avoid overflowing in the case of clogging of the CBC media and to provide a uniform flow across the cross-sectional area of each column. This strategy of upward flow was consistent with the study by Mjengera and Mkongo, where cow bone char was used to remove fluoride from water. The authors mentioned that very fine particles are not recommended for use in column experiments because of the tendency of fine particle sizes to clog [3]. In our study, we used a fine particle size of CBC 106–212 µm in diameter in columns with regular ultrasonication to successfully avoid clogging. The experiment was done at temperatures between 20 and 27 °C.

Chemical composition analysis, X-ray diffraction (XRD) pattern analysis, scanning electron microscope (SEM) analysis, and Brunauer–Emmett–Teller (BET) surface area analysis were conducted for the 106–212 µm CBC used for the study. CBC particles before the fluoride adsorption were analyzed as a control.

The chemical composition of CBC and the solutions were analyzed using ion chromatography (for anions: Dionex ICS-2000, separation column IonPac AS18, eluent KOH 23–40 mmol/L (gradient), suppressor ASRS 300 4 mm; for cations: Dionex ICS-1500, separation column IonPac CS12, eluent methanesulfonic acid 30 mmol/L (isocratic), suppressor CSRS 500 4 mm). The chemical composition of CBC was analyzed after digesting the samples. Samples (0.1 g) were digested with 5% nitric acid to analyze the anions and cations other than F^−^ in CBC. The F^−^ in CBC was determined after the steam distillation. The following were kept at 418 K in a flask: 0.1 g of CBC, 40 mL of HClO_4_, 1 g of SiO_2_, 1 mL of H_3_PO_4_, and 10 mL of distilled water. Steam was introduced into the flask to vaporize F^−^. The vaporized F^−^ was condensed with steam in a water-jacketed condenser for measurement by ion chromatography. The carbon (C), hydrogen (H), nitrogen (N), and carbonate (CO_3_^2−^) contents of CBC were analyzed using a CHN Corder (Yanaco, MT-5). The CO_3_^2−^ content of CBC was analyzed after heating chicken bones to 973 K under an aerobic condition in an electrical muffle furnace to remove organic carbon. The XRD of hydroxyapatite (HAP) and the CBC was analyzed using MiniFlex (Rigaku Co., Tokyo, Japan) X-ray 30 kV/15 mA, radiation CuK alpha line (Ni filter), scintillation detector. SEM images of the CBC were taken using a VE-8800 (Keyence Co., Osaka, Japan). The BET surface area of the CBC was analyzed using the NOVA 3200e Surface Area and Pore Size Analyzer (Quantachrome Instruments, Boynton Beach, FL, USA).

## 3. Results and Discussion

### 3.1. Selecting the Best Carbonizing Temperature for the Preparation of CBC

**Figure 2:**
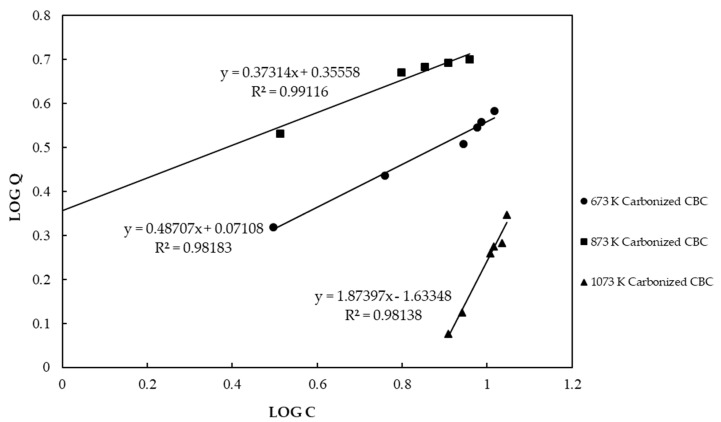
Freundlich isotherm for chicken bone char (CBC) carbonized at different temperatures.

When preparing CBC, selecting the appropriate carbonizing temperature is the most important factor as it affects the efficacy of CBC in removing fluoride from contaminated water. Figure 2 shows the Freundlich isotherm for CBC carbonized at 673 K, 873 K, and 1073 K. In the figure, C is fluoride concentration in the solution expressed in a unit of mg/L, while Q is maximum adsorption capacity expressed in a unit of mg/g.

The F^−^ adsorption by CBC carbonized at three temperatures matched well with the Freundlich isotherm. According to the Freundlich isotherm, CBC carbonized at 673 K, 873 K, and 1073 K showed fluoride adsorption capacities of 3.62 mg/g, 5.35 mg/g, and 1.74 mg/g, respectively, at the equilibrium fluoride concentration of 10 mg/L. It was obvious that chicken bones carbonized at 873 K showed the highest adsorption capacity. Further, we observed a yellowish color, undesirable taste, and unpleasant odor in water treated with chicken bones carbonized at 673 K. Therefore, we selected 873 K as the best carbonizing temperature for further experiments.

The results obtained from our study were consistent with the literature. When the carbonizing temperature decreases to less than 773 K, odor, undesirable taste, and yellowish color occur in the treated water due to less (or no) removal of organic matter such as fats, oil, and meat residuals [3] from chicken bones. Leyva-Ramos and his fellows had reported that carbonizing temperatures below 773 K caused an unpleasant taste and smell and yellowish color in treated water due to the organic matter in bone char [21], similar to what we found in our study. We detected that the weight-basis C content in chicken bones carbonized at 673 K (14%) was higher than the C content in CBC carbonized at 873 K (9%) and 1073 K (6%), showing that there was considerably more organic matter in CBC carbonized at 673 K. At lower carbonizing temperatures (573 K), the organic matter in bones was insufficiently removed, which made bones unable to provide a large specific surface area and enough pore space for the efficient removal of fluoride [22].

When the carbonizing temperature increased to more than 873 K, the adsorption capacity of CBC was reduced dramatically. Carbonizing temperatures higher than 873 K can alter the hydroxyapatite structure of bone [22], leading to a reduction in fluoride removal capacity [23], as we found in our study. Kawasaki et al. investigated the fact that the adsorption capacity of fluoride onto bone char carbonized at 1073 K for 2 h was higher than that carbonized for 2 h at 1273 K by studying four types of bone char: cow bone char, pig bone char, chicken bone char, and fish bone char [24]. Mayorga et al. reported that the best fluoride removal performance of bone char was observed at the carbonizing temperature of 973 K for 2 h by studying cow bone char. Furthermore, they mentioned that when the carbonizing temperature increased to more than 1073 K, the fluoride ion adsorption capacity of bone char decreased from 6.0 mg/g to 3.0 mg/g, and when increasing the carbonizing temperature to 1273 K, the fluoride adsorption capacity was reduced to 1.0 mg/g. Finally, using their results, they justified that the carbonizing temperature of bone char plays a major role in water defluoridation [6]. We selected 873 K as the best carbonizing temperature considering the adsorption capacity and the energy consumption.

### 3.2. Adsorption Capacity of the CBC Based on Equilibrium Sorption Isotherms

**Figure 3:**
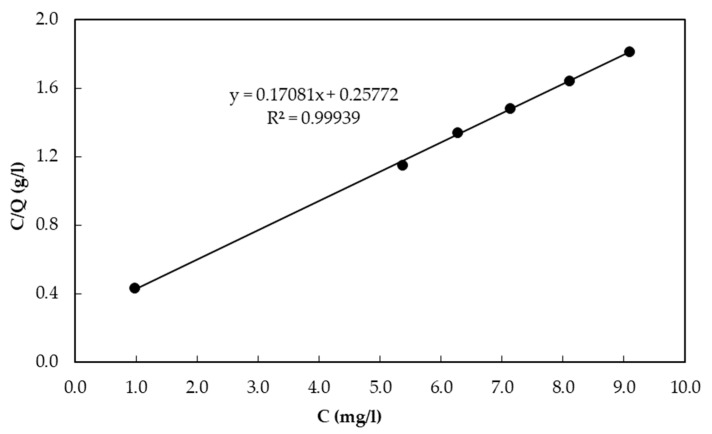
Langmuir isotherm for CBC.

Freundlich and Langmuir isotherm models were evaluated to investigate the adsorption capacity of CBC. Figure 2 shows the Freundlich isotherm for CBC. Figure 3 shows the Langmuir isotherm for CBC. The Langmuir isotherm model was found to be better fitted than the Freundlich isotherm model. According to the Freundlich and Langmuir isotherms, the adsorption capacity of CBC at the equilibrium fluoride concentration of 10 mg/L was 5.35 mg/g and 5.88 mg/g, respectively.

### 3.3. Fluoride Removal Mechanism of CBC

**Figure 4:**
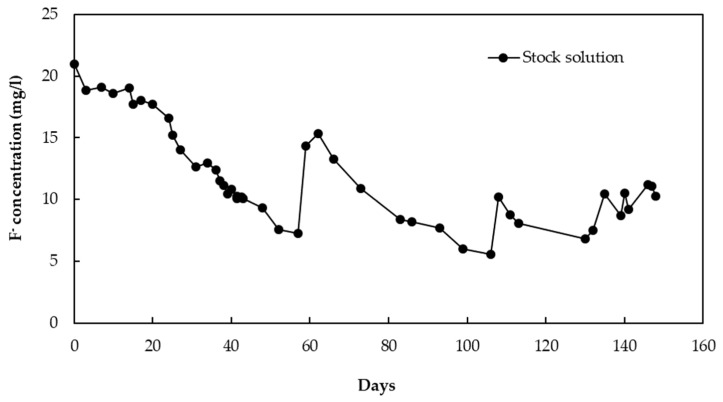
Change in the fluoride concentration of the stock solution.

Figure 4 shows change in the fluoride concentration of the stock solution throughout the operation period. Table 1 gives a detailed description of the amount of F^−^ in the solution throughout the operation period. A certain amount of F^−^ was lost from the stock solution due to leakage, and this was considered when calculating the adsorption capacity. The CBC particles with a diameter of 106–212 µm showed an adsorption capacity of 11.2 mg/g at a fluoride concentration of 10 mg/L after the operation period of 148 days. The adsorption capacity was calculated according to the data in Table 1 (1855 mg F^−^/(55*3) g CBC).

It is obvious that the CBC showed an unusually high adsorption capacity, nearly 2 times the adsorption capacity obtained by the Langmuir isotherm, indicating that equilibrium was not established within 24 h. To confirm the unusual fluoride adsorption capacity, the fluoride content in the CBC before and after 148 days of operation was measured using steam distillation.

Table 2 shows the averaged value of the adsorption capacities of F1, F2, and F3 after the adsorption. An adsorption capacity of 11.1 mg/g coincided well with the adsorption capacity obtained from the mass balance calculation for the solution, as shown in Table 1.

According to studies relating to the fluoride adsorption capacity of bone char, CBC with a particle size >0.075 mm, 0.075–0.30 mm, 0.30–1.18 mm, and 1.18–2.34 mm showed a fluoride adsorption capacity of 0.665 mg/g, 0.661 mg/g, 0.660 mg/g, and 0.643 mg/g, respectively, at the equilibrium fluoride concentration of 10 mg/L [25]. They also reported the fluoride adsorption capacity of lamb bone char with particle sizes >0.075 mm, 0.075–0.30 mm, 0.30–1.18 mm, and 1.18–2.34 mm for which the fluoride adsorption capacity was 0.482 mg/g, 0.475 mg/g, 0.459 mg/g, and 0.414 mg/g, respectively, at the equilibrium fluoride concentration of 10 mg/L [25].

In a study using 0.79 mm cattle bone char particles, a fluoride adsorption capacity of 2.71 mg/g was recorded at the equilibrium fluoride concentration of 1 mg/L [7]. Rojas-Mayorga and his fellow researchers showed a fluoride adsorption capacity of 7.32 mg/g using ~1 mm cow bones at the equilibrium fluoride concentration of 60 mg/L [6].

No studies have reported such an unusually high fluoride adsorption capacity of bone char.

**Figure 5:**
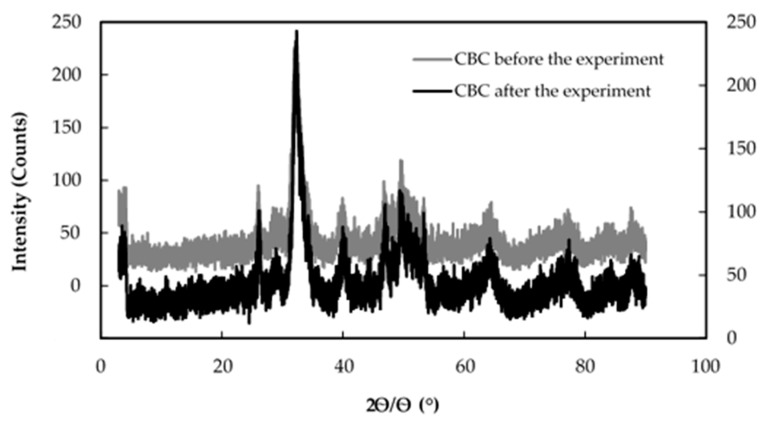
X-ray diffraction patterns of CBC before and after the fluoride adsorption. CBC before the experiment (left *Y* axis), CBC after the experiment (right *Y* axis).

Figure 5 shows the X-ray diffraction patterns of CBC before and after the fluoride adsorption.

According to Figure 5, it is clear that the two X-ray diffraction patterns of CBC before and after the fluoride adsorption overlapped, showing that the crystal structure of CBC before and after the fluoride adsorption was similar.

It has been reported that fluoride removal by bone char is a surface reaction process [22]. Table 3 shows the BET surface area of CBC before and after the fluoride adsorption.

According to Table 3, the finer particle size of CBC 106–212 µm before and after the fluoride adsorption showed a similar surface area.

**Figure 6:**
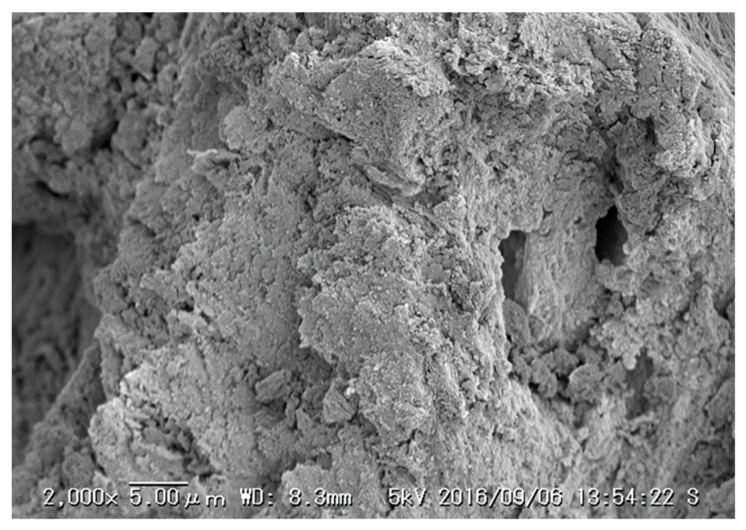
SEM image of the CBC before fluoride adsorption.

**Figure 7:**
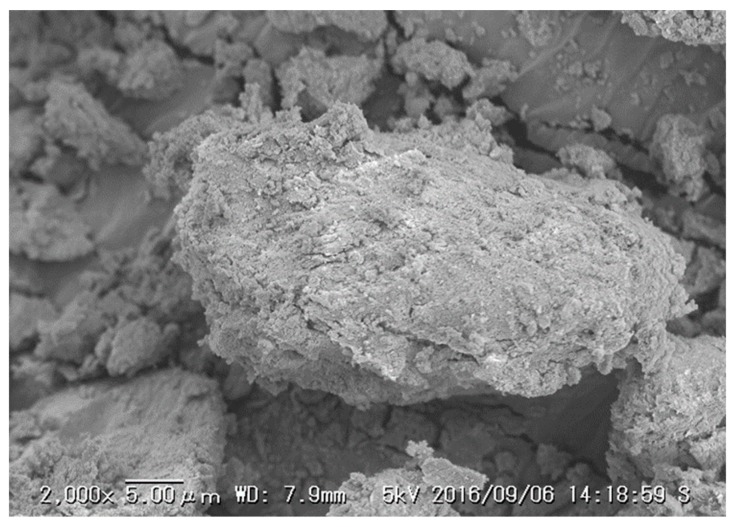
SEM image of the CBC after fluoride adsorption.

SEM images of 106–212 µm CBC used for the study were taken in two different stages to compare the surface, morphology, and size distribution. Figure 6 and Figure 7 show SEM images of the CBC before and after fluoride adsorption, respectively.

The SEM images in Figure 6 and Figure 7 show similar structures of CBC, as evident from the similar X-ray diffraction patterns of CBC before and after the fluoride adsorption in Figure 5. This was further confirmed by the almost-equal surface area of CBC before and after the fluoride adsorption, as shown in Table 3.

It has been reported in the literature that fluoride removal by bone char (CBC) is associated with the two main mechanisms of ion exchange and chemical precipitation. In the presence of fluoride ion, the hydroxyl ion in HAP is replaced by fluoride ion to form insoluble fluorapatite (FAP) [25] and release the hydroxyl ion into the solution. F^−^ and OH^−^ consist of the same charge and a similar size of radius. Therefore, the fluoride ion can replace the hydroxyl ion in mineral structures [22].

The relevant chemical reaction can be represented as Equation (1) [26]:

Ca_10_(PO_4_)_6_(OH)_2_ + 2F^−^ → Ca_10_(PO_4_)_6_F_2_ + 2OH^−^ (1)

In the presence of an excess fluoride ion, HAP precipitates into calcium fluoride (CaF_2_), and the phosphate in HAP is released into the solution. 

The relevant chemical reaction can be represented as Equation (2) [22]:

Ca_10_(PO_4_)_6_(OH)_2_ + 20F^−^ + 2H^+^ → 10CaF_2_ + 6PO_4_^3−^ + 2H_2_O (2)

According to the similar XRD patterns, SEM images, and BET surface area of CBC, there was no evidence indicating that the formation of CaF_2_ took place.

Table 4 shows the anion and cation concentrations of the solution before and after the adsorption. A detailed description of the fluoride concentration in the solution is given in Table 1.

According to Equation (2), the phosphate in HAP should be released into the solution with the formation of CaF_2_. On the contrary, there was no evidence of phosphate in the solution.

According to the solubility product constant (Ksp) of CaF_2_ and the molar concentrations of Ca^2+^ and F^−^ in the final solution, there was a possibility that CaF_2_ precipitated in the solution due to the reaction of F^−^ in the solution and released Ca^2+^ from the CBC to the solution. The Ksp of CaF_2_ (3.4 × 10^−11^ mol^3^/L^3^) was calculated from the solubility of CaF_2_ (0.016 g/L in water at 20 °C). The molar concentration of Ca^2+^ and F^−^ in the final solution was calculated as 2.7 × 10^−10^ mol^3^/L^3^, which exceeded the Ksp value. However, there was no visible CaF_2_ precipitation in the experimental setup.

**Figure 8:**
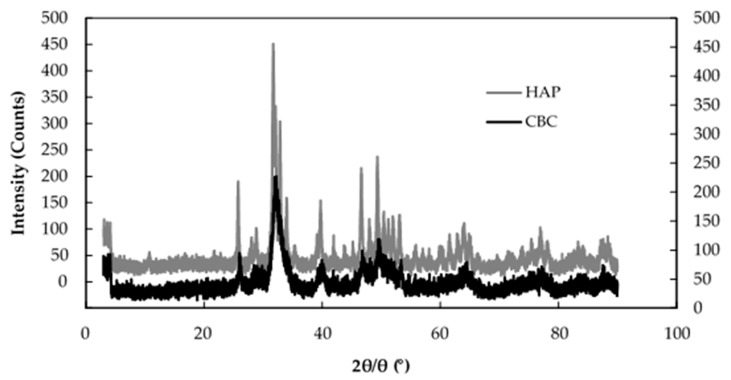
XRD patterns of HAP and CBC. Hydroxyapatite (HAP) (left *Y* axis), CBC (right *Y* axis).

Figure 8 shows the XRD patterns of HAP and CBC. Their similar patterns indicate that the major component of CBC was HAP. Table 5 shows the number of moles of PO_4_^3−^, Ca^2+^, F^−^, and OH^−^ in 100 g of CBC before and after the fluoride adsorption based on chemical analysis. The Ca^2+^/PO_4_^3−^ molar ratio of 1.86 for CBC (before the fluoride adsorption) was similar to that of 1.67 for hydroxyapatite: [Ca_10_(PO_4_)_6_(OH)_2_] (HAP).

The number of moles of OH^−^ in CBC before the fluoride adsorption was calculated based on the molar ratio of Ca^2+^:OH^−^ (10:2) before the fluoride adsorption, assuming that the major component of CBC is hydroxyapatite. The number of moles of OH^−^ in CBC after the fluoride adsorption was calculated based on the molar ratio of Ca^2+^:OH^−^ (10:2) and by reducing the F^−^ moles.

According to the chemical composition, CBC contained 65.3% HAP and 9% C on a weight basis. The percentage of HAP in the CBC was calculated by the sum of the percentages of Ca^2+^ (27.7%) and PO_4_^3−^ (35.2%) in the CBC digested with nitric acid and OH^−^ (2.4%), which was calculated from the molar ratio of Ca^2+^:OH^−^ (10:2). The result obtained in our study was consistent with the literature. Brunson and Sabatini and Abe et al. have mentioned that bone char contains approximately 75% hydroxyapatite [Ca_10_(PO_4_)_6_(OH)_2_], 9–11% calcite (CaCO_3_) [22], and 8–10% C [27]. Further, we could detect 0.6% Mg^2+^, 0.5% Na^+^, 0.1% K^+^, 0.1% Cl^−^, 1.3% N, and 0% CO_3_^2−^ on a weight basis as trace components in the CBC. Ooi et al. also reported that Ca and P are the major components in bone char and that Na, Mg, O, and C are minor components in bone char based on their study of bovine bone char [28].

The increase of Na^+^ in the final solution (Table 4) was mainly due to the addition of NaF to the solution to maintain the fluoride concentration. Cl^−^, K^+^, Mg^2+^, and Ca^2+^ ions, which were not present in the initial solution, were detected in the final solution (Table 4) after 148 days of operation. This was due to the dissolution of those ions in the final solution from CBC as we detected them as components in CBC. A certain amount of Na^+^ may also have been released into the solution by the dissolution from CBC as we also detected Na^+^ as a trace component in CBC.

When the reaction of Equation (1) is taken into consideration, a certain amount of HAP was converted to FAP. Considering the number of OH^−^ moles in CBC before and after the experiment in Table 5, 45.6% of HAP could be converted to FAP. According to Equation (1), the same molar of OH^−^ should be released into the solution; however, a significant change in pH value was not observed. Table 6 shows the pH, number of OH^−^ moles, and alkalinity of the solutions.

The total amount of F^−^ removal was 97.6 mmol. This was calculated according to the data in Table 1 (1855 mg/19 g/mol). The released OH^−^ could be partly neutralized by CO_2_ dissolved from the atmosphere to produce alkalinity as we detected 762 μeq/L of alkalinity in the final solution.

In relation to the high adsorption by bone char, Mwaniki reported that Cl^−^ ions increased the rate of fluoride adsorption onto bone charcoal [29]. Abe et al. also reported that fluoride adsorption by bone char increased in the presence of Cl^−^ ions in the solution. They discussed the “salting out” effect of NaCl that is relevant to the excess fluoride adsorption by bone char. NaCl dissociates in water by giving Na^+^ and Cl^−^ ions to the solution. Na^+^ and Cl^−^ ions in the solution are hydrated with water molecules by reducing the water molecules for the dissolution of fluoride. Therefore, the fluoride ion in the solution is enhanced to be adsorbed onto bone char [27].

**Figure 9:**
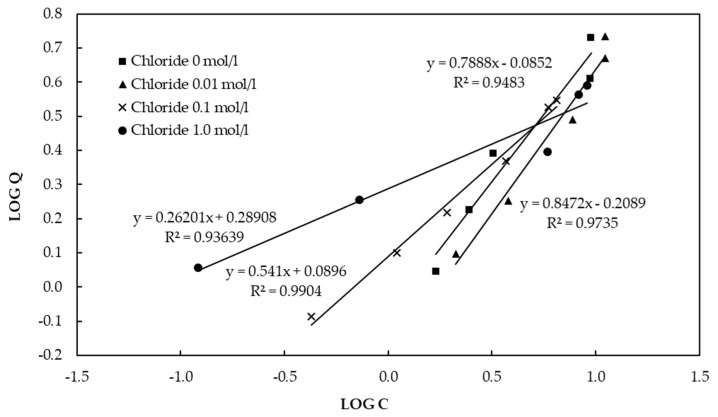
Freundlich isotherm for CBC in the presence of chloride.

In contrast, our experiment showed that higher Cl^−^ concentrations decreased the fluoride adsorption capacity of CBC. Figure 9 shows the Freundlich isotherm for CBC in the presence of chloride. According to the Freundlich isotherm, the adsorption capacities of CBC in the presence of Cl^−^ concentrations of 0 mol/L, 0.01 mol/L, 0.1mol/L, and 1.0 mol/L were 5.1 mg/g, 4.4 mg/g, 4.3 mg/g, and 3.6 mg/g, respectively, at a fluoride concentration of 10 mg/L. According to Table 4, Cl^−^ ions were slightly released from CBC to the solution as 0.002 mol/L was detected in the final solution. The release of Cl^−^ from CBC to the solution caused a decrease in the adsorption of fluoride onto the CBC. Consequently, “salting out” was not the reason for the excess adsorption of fluoride.

## 4. Conclusions

Defluoridation of drinking water is essential in order to avoid potential human health risks from fluoridated water. Finer-sized CBC was investigated for fluoride removal from drinking water in relation to its adsorption capacity using a column experiment. Results showed an unusual adsorption capacity of 11.2 mg F^−^/g CBC, which was higher than that have been previously reported. The XRD patterns, SEM images, and BET surface area of CBC showed that the structure of CBC before and after the fluoride adsorption was similar. The smaller radius of finer-sized CBC enhanced the mechanism of fluoride adsorption by ion exchange.

## Figures and Tables

**Table 1:** Amount of F^−^ in the solution.

F^−^ in the Solution	mg
F^−^ added to the solution	2831
F^−^ lost from the solution by leakage	492
F^−^ remaining in the solution	483
F^−^ adsorbed by CBC (55 g*3)	1855

**Table 2:** Averaged value of adsorption capacities of F1, F2, and F3.

F^−^ Adsorption Capacity (mg/g)				
F^−^ Content in CBC before Adsorption	F^−^ Content in CBC after Adsorption		Net Adsorption	Averaged Value
0.46	F1	12.13	11.67	11.1
	F2	12.05	11.59	
	F3	10.43	9.97	

**Table 3:** BET surface area of CBC before and after the fluoride adsorption.

Particle Size	State of CBC	BET Surface Area (m^2^/g)
106–212 μm	CBC before the fluoride adsorption	126
	CBC after the fluoride adsorption	136

**Table 4:** Anion and cation concentrations of solution used for the experiment.

Solution	Concentration (mg/L)							
	F^−^	Cl^−^	PO_4_^3−^	Na^+^	NH_4_^+^	K^+^	Mg^2+^	Ca^2+^
Initial solution	20	0	0	19	0	0	0	0
Final solution	10	87	0	61	0	3	5	10

**Table 5:** Number of moles of PO_4_^3−^, Ca^2+^, F^−^, and OH^−^ in CBC before and after the fluoride adsorption.

State of CBC	No. of Moles in 100 g of CBC			
	PO_4_^3−^	Ca^2+^	F^−^	OH^−^
CBC before the fluoride adsorption	0.371	0.691	0.002	0.138
CBC after the fluoride adsorption	0.351	0.684	0.061	0.075

**Table 6:** Solution pH, number of OH^−^ moles, and alkalinity.

Solution	pH	No. of OH^−^ Moles (µ moles/L)	Alkalinity (μeq/L)
Initial solution	5.21	0.002	0
Final solution	7.82	0.661	762
